# Prevalence and correlates of current use of heated tobacco products among a nationally representative sample of Korean adults: Results from a cross-sectional study

**DOI:** 10.18332/tid/125232

**Published:** 2020-08-05

**Authors:** Seung Hee Kim, Hong-Jun Cho

**Affiliations:** 1 Department of Family Medicine, Asan Medical Center, University of Ulsan College of Medicine, Seoul, Republic of Korea

**Keywords:** tobacco products, prevalence, association, prevention

## Abstract

**INTRODUCTION:**

Heated tobacco product (HTP) use has increased rapidly in Korea since its introduction in 2017. We investigated the prevalence and correlates of the current use of HTPs among a nationally representative sample of Korean adults.

**METHODS:**

A total of 6182 participants aged ≥19 years took part in the 2018 Korea National Health and Nutrition Examination Survey conducted one year after the introduction of HTPs in Korea. Descriptive statistics, bivariate analysis and multivariable logistic regressions were used.

**RESULTS:**

The prevalence of current HTP use (defined as past 30-day use) among Korean adults was 4.4% (7.8% for males and 0.9% for females) in 2018. Among current HTP users, approximately 90% were dual users with combustible cigarettes (CCs) or electronic cigarettes (ECs), or triple users with CCs and ECs. In multivariable logistic regression analysis, males, younger participants, and current CC and/or EC users showed greater odds of being current users of HTPs compared with females, older participants, and non-users of CCs and ECs. Moreover, current EC-only users were more likely to use HTPs than current CC-only users. Compared with current CC-only use, using HTPs with CCs concurrently was not associated with attempts to quit smoking during the past year or with intentions to quit CC smoking.

**CONCLUSIONS:**

The popularity of HTP use and the pattern of its poly use with CCs and ECs is a new challenge for Korean tobacco control policy.

## INTRODUCTION

Heated tobacco products (HTPs) release aerosols containing nicotine and toxic chemicals upon heating of the tobacco without combustion^1^. IQOS was introduced in 2014 and is now available in 51 countries^2^. HTPs were introduced in Korea in June 2017 and their market share increased rapidly; in 2019, they accounted for 10.5% of the total tobacco in the country^3^.

The rate of current HTP use was 5.0% among Japanese adults in 2018^4^. In 2017, 1.4% of Italian adults were ever users of HTPs^5^. However, in countries where their sale started later, the proportion of adult users was lower. As of 2017, there were 0.8% current users in Great Britain^6^ and 1.1% in the United States^7^, where IQOS had been available since 2016 and 2019, respectively. In Korea, the prevalence of HTP use was 3.5% in 2017^8^, and 2.13% in 2018^9^.

Philip Morris International claimed that switching completely from combustible cigarettes (CCs) to IQOS would reduce health risk^10^. However, most HTP users used CCs or/and electronic cigarettes (ECs) rather than switching to HTPs completely^6-9^. According to independent studies, the levels of some harmful and potentially harmful constituents of HTPs were lower than those of CCs^10,11^. Nevertheless, the long-term effects of HTPs on individual- or population-level health are yet to be determined. According to a recent study, CC users who had used HTPs were significantly less likely to be former CC users despite having made more attempts at smoking cessation^12^.

CC or EC use was the most significant factor associated with HTP use^7-9^ and participant characteristics such as being male, being younger, and having higher education and economic status were correlated to its use^8,9^. HTP use was not related to having made attempts at or having intentions of smoking cessation^9,13^. Prior Korean studies covered one province of Korea^9^ or dealt with young adults only^8^. The present study demonstrated the prevalence and correlates of HTP use and identified whether HTP use was associated with having made attempts at or having intentions of smoking cessation among a nationally representative sample of Korean adults.

## METHODS

### Data and study participants

We collected data from the 2018 Korea National Health and Nutrition Examination Survey (KNHANES), a nationally representative survey conducted annually. This survey applied a stratified multistage sampling design to extract representative samples. A detailed description of the KNHANES is provided in Kweon et al.^14^. The overall response rate was 76.5% and the final sample included 6182 adults aged ≥19 years, after excluding 306 incomplete questionnaires.

### Measures

Never CC users were defined as those who answered ‘no’ or ‘ <100 cigarettes’ to the question: ‘How many cigarettes have you smoked?’. Current CC users were defined as those who had a smoking history of ≥100 cigarettes and answered ‘every day’ or ‘some days’ to the question: ‘Do you smoke cigarettes?’. Current CC-only users were classified as: non-daily CC users, light daily CC users (<10 cigarettes/day), moderate daily CC users (10–19 cigarettes/day), and heavy daily CC users (≥20 cigarettes/day). Former CC users were defined as those who had smoked at least 100 cigarettes but did not smoke cigarettes now. Never EC users were those who answered ‘no’ to the question: ‘Have you ever used e-cigarettes?’. Current EC users included those who answered ‘yes’ to the question: ‘Have you used ECs during the past 30 days?’. Former EC users included those who were ever users but had not used ECs in the past 30 days. Ever HTP users included those who checked ‘heated tobacco’ to the question: ‘Check all the products you have ever used: 1=snus, 2=waterpipes, 3=cigars, 4=heated tobacco (IQOS, Glo, etc.), 5=other, and 6=none’. Current HTP users included those who checked ‘heated tobacco’ to the question: ‘Check all the tobacco products you have used during the past 30 days’. Sociodemographic characteristics included sex, age, educational level, and household income.

CC quit attempters were defined as CC users who answered ‘yes’ to the question: ‘During the past year, have you made attempts to quit smoking for more than a day?’. Intentions to quit smoking were evaluated based on whether they had had intentions on smoking cessation within 1 month.

### Statistical analysis

All analyses were considered survey weights for the complex sampling design of the 2018 KNHANES. We conducted bivariate testing and multivariable logistic regression analyses to evaluate the prevalence, correlates of HTP use, and the associations between HTP use and attempts, as well as intentions to quit smoking. All statistical analyses were done using IBM SPSS Statistics for Windows, Version 24.0.

## RESULTS

The prevalence of current HTP users was 4.4% (7.8% males and 0.9% females): 6.8% among those aged 19–34 years, 7.9% among those aged 35–49 years, and 0.6% among those aged ≥50 years. Among those who had completed college, the rate of HTP users was 6.5%. However, for those with low education levels, it was 0.8%. Prevalence of HTP users was higher in those who had a high income (5.7%) than those who had a low income (2.4%). Current HTP-only users were all former CC or EC users. The rate of HTP use was much higher for CC-only users (8.4%), EConly users (53.3%), and dual users of CCs and ECs (68.0%) than non-users (0.6%) (Table 1).

**Table 1:** Prevalence and correlates of the current use of HTPs, and the association between HTP and/or EC use, quit attempts, and intentions to quit CC smoking (N=6182)

*Characteristics*	*Full sample*	*Current use of IITPs*			*Current CC users*	*Attempted to quit smoking*		*Intends to quit smoking*	
		*Bivariate analysis*	*Multivariable analysis*			*Bivariate analysis*	*Multivariable analysis*	*Bivariate analysis*	*Multivariable analysis*

	*n*	*n %*	*AOR ^b^ (95% CI)*	*AOR ^c^ (95% CI)*	*n*	*n (%)*	*AOR ^d^ (95% CI)*	*n (%)*	*AOR ^d^ (95% CI)*
**Total**	6182	205 (4.4)							
**Sex**									
Female	3469	28 (0.9)	1						
Male	2713	177 (7.8)	**8.93 (5.96-13.36)**						
**Age** (years)									
>50	3385	17 (0.6)	1						
35-49	1669	113 (7.9)	**13.26 (6.42-27.39)**						
19-34	1128	75 (6.8)	**11.19 (5.16-24.27)**						
**Education**									
< Middle school graduate	1748	7 (0.8)	1						
High school graduate	1990	72 (4.5)	1.15 (0.40-3.30)						
> College graduate	2215	123 (6.5)	1.38 (0.50-3.85)						
**Household income**									
1st quartile (lowest)	1179	16 (2.4)	1						
2nd quartile	1486	46 (3.6)	0.77 (0.42-1.41)						
3rd quartile	1682	61 (4.7)	0.86 (0.46-1.62)						
4th quartile (highest)	1817	81 (5.7)	1.07 (0.58-1.98)						
**CC and EC use status**									
Non-users of CCs and ECs	5042	25 (0.6)^a^		1					
Current CC-only users (daily)	969	70 (8.4)							
Non	141	11 (7.3)		**9.01 (3.96-20.53)**					
Light (<10 cigs/day)	133	7 (6.9)		**10.11 (3.96-25.80)**					
Moderate (10-19 cigs/day)	401	39 (11.2)		**13.11 (7.04-24.42)**					
Heavy (>20 cigs/day)	294	13 (5.7)		**6.45 (2.52-16.52)**					
Current EC-only users	19	10 (53.3)		**88.46 (22.43-348.91)**					
Dual users of CCs and ECs	152	100 (68.0)		**222.54 (109.90-450.62)**					
**Current CC use status**									
All					1121				
CC-only					899	483 (52.9)	1	167 (18.1)	1
CCs + ECs					52	31 (63.4)	1.48 (0.77-2.86)	10 (19.0)	1.22 (0.54-2.74)
CCs + HTPs					70	32 (45.1)	0.60 (0.34-1.06)	5 (6.1)	0.39 (0.15-1.04)
CCs + ECs + HTPs					100	49 (50.1)	0.83 (0.48-1.42)	12 (11.2)	0.69 (0.31-1.56)

Values are presented as unweighted numbers (weighted percentages). Bolded values indicate significance at p<0.05. AOR: adjusted odds ratio; CI: confidence interval; HTP: heated tobacco product; CC: combustible cigarette; EC: electronic cigarette; Cigs: cigarettes.

a Current HTP-only users were all former CC or EC users.

b Adjusted for sex, age, education, and household income.

c Adjusted for sex, age, education, household income, and CC and EC use status.

d Adjusted for sex, age, education, household income, and current CC use status.

In multivariable logistic regression analysis for correlates of HTP use, the odds of using HTPs were 8.93 (95% CI: 5.96–13.36) for males compared to females, 11.19 (95% CI: 5.16–24.27) for those aged 19–34 years, and 13.26 (95% CI: 6.42–27.39) for those aged 35–49 years, compared to those aged ≥50 years. Odds of HTP use were 9.01 (95% CI: 3.96–20.53) for non-daily smokers, 10.11 (95% CI: 3.96–25.80) for light smokers, 13.11 (95% CI: 7.04–24.42) for moderate smokers, 6.45 (95% CI: 2.52–16.52) for heavy smokers, 88.46 (95% CI: 22.43–348.91) for EC-only users, and 222.54 (95% CI: 109.90–450.62) for dual users of CCs and ECs compared with non-users of CCs and ECs.

In multivariable logistic regression analysis on CC users, the odds of attempts to quit smoking were 0.60 (95% CI: 0.34–1.06) for dual users of CCs and HTPs, and 0.83 (95% CI: 0.48–1.42) for triple users compared with CC-only users. Odds of intentions to quit smoking were 0.39 (95% CI: 0.15–1.04) for dual users of CCs and HTPs, and 0.69 (95% CI: 0.31–1.56) for triple users compared with CC-only users.

## DISCUSSION

The prevalence of current HTP users among Korean adults reached 4.4.% a year after its introduction in Korea—a much higher rate than those of Italy, United States, or Great Britain, but similar to Japan’s^4-7^. Although this confirmed that HTPs are relatively more popular in East Asian countries, it should be interpreted cautiously, as their introduction time is different for various countries. In Korea, HTPs are regulated the same way as CCs, except for the tobacco tax; both smoking and using HTPs in public places are banned, and it is mandatory to put pictorial warning labels on the packages of CCs and HTPs. Nevertheless, the popularity of HTPs in a short period suggests a successful marketing strategy by companies in Korea, which, combined with HTPs’ characteristic of less odor than CCs, helped attract smokers^15^.

Most HTP users were dual or triple users of CCs and/or ECs, which was consistent with prior studies^5,7-9^. HTP use was not associated with attempts and intentions to quit smoking in line with previous studies^7,9,13^. These imply that HTPs were not being used as a tool for quitting smoking and that HTPs were more likely to be used concurrently with CCs as another method of consuming tobacco. Although the current HTP-only users were all former CC or EC users, it was not evident whether HTPs helped tobacco users quit CCs or ECs, or led to quitters of CCs or ECs becoming HTP users. A further longitudinal study is needed to explain this.

The odds of HTP use were much greater in EC users than in CC users, as with prior studies^7,9,12^. Among CC-only users, moderate smokers were more likely to use HTPs as in a previous study^9^. Because nicotine dependence was associated with HTP use among smokers^13^, it is likely that the degree of nicotine dependence in moderate smokers was most associated with HTP use. Heavy daily smokers were less likely to use HTPs among smokers, which can be explained by characteristics of HTPs such as lower level of nicotine delivery than CCs and high cost^9,15^. The reason behind the odds of HTP use of EC users being greater than those of CC users was the many similarities between them. Both are marketed as being less harmful and as having benefits of reduced ash and odor^15^. The use of HTPs was higher among males and younger age, which can be explained by the marketing strategy of tobacco companies targeting young adults^15^, alongside a higher prevalence of tobacco use among males than females.

### Limitations

There were limitations. As this study was cross-sectional, causal relationships could not be identified. The questions for HTP use were different from those of CC and EC use, which may have led to biased responses regarding the prevalence of HTP use. The reason for using HTPs was not assessed in the survey.

## CONCLUSIONS

About 4.4% of the Korean adult population were current HTP users in 2018, with most being dual and triple users of CCs or/and ECs. We need to carefully monitor the use of HTPs, CCs, and ECs and formulate tobacco control policies to cope with the increasing popularity of HTPs.
